# Synthesis and structure of 1,1′,1′′-[(2-bromo­eth­oxy)methane­tri­yl]tri­benzene and 1,1′,1′′-[(2-iodoeth­oxy)methane­tri­yl]tri­benzene

**DOI:** 10.1107/S2056989025001124

**Published:** 2025-02-14

**Authors:** Julian Fischer, Marian Hebenbrock

**Affiliations:** a Institut für Anorganische und Analytische Chemie, Corrensstr. 28/30, 48149 Münster, Germany; University of Massachusetts Dartmouth, USA

**Keywords:** crystal structure, trityl group, haloalkanes

## Abstract

The present study reports on the mol­ecular structures and syntheses of 1,1′,1′′-[(2-bromo­eth­oxy)methane­tri­yl]tri­benzene, C_21_H_19_BrO, and 1,1′,1′′-[(2-iodo­eth­oxy)methane­tri­yl]tri­benzene, C_21_H_19_IO. Both compounds crystallized from a saturated solution in THF by slow vapour diffusion of *n*-hexane in the monoclinic space group *P*2_1_/*c*. While the two independent mol­ecules in the asymmetric unit of 1,1′,1′′-[(2-iodo­eth­oxy)methane­tri­yl]tri­benzene show no close contacts to other mol­ecules, in the structure of 1,1′,1′′-[(2-bromo­eth­oxy)methane­tri­yl]tri­benzene the mol­ecules inter­act with each other *via* C—H⋯π contacts.

## Abstract 2

Both compounds crystallized from a saturated solution in THF by slow vapour diffusion of *n*-hexane in the monoclinic space group *P*2_1_/*c*. While the two independent mol­ecules in the asymmetric unit of 1,1′,1′′-[(2-iodo­eth­oxy)methane­tri­yl]tri­benzene show no close contacts to other mol­ecules, in the structure of 1,1′,1′′-[(2-bromo­eth­oxy)methane­tri­yl]tri­benzene the mol­ecules inter­act with each other by C—H⋯π contacts.

## 1. Chemical context

2-Haloethyl trityl ethers are bifunctional compounds that combine halogen reactivity with trityl ether protection for controlled synthesis. Alkyl halides, as a class of versatile inter­mediates in organic chemistry, facilitate the formation of a broad array of carbon–carbon bonds and functional group transformations. The polarized carbon–halogen bond facilitates substitution and elimination reactions, with the extent of reactivity being influenced by factors such as the halogen type, the alkyl group, and the reaction conditions. Consequently, these compounds are valuable in diverse synthetic reactions. Alkyl halides with a trityl ether group are widely employed as alkyl­ating agents in biomedical chemistry for drug development (Gunosewoyo *et al.*, 2013 ▸; Wagner *et al.*, 2009 ▸; Sureshan *et al.*, 2001 ▸; Smits, 2006 ▸). For instance, 1,1′,1′′-[(2-bromo­eth­oxy)methane­tri­yl]tri­benzene has been used in the development of selective inhibitors for serine/threonine kinases (Gunosewoyo *et al.*, 2013 ▸; Wagner *et al.*, 2009 ▸). 1,1′,1′′-[(2-Iodo­eth­oxy)methane­tri­yl]tri­benzene was employed as an alkyl­ating agent in the research conducted for the total synthesis of (–)-kendomycin (Smits, 2006 ▸). The trityl protecting group provides selective protection under basic conditions and can be readily cleaved under mild acidic conditions. The trityl group and its derivatives, such as dimeth­oxy trityl (DMT), are commonly used in the synthesis of nucleotides and peptides, where they serve as a protecting group for hydroxyl or amino groups, enabling selective reactions without inter­ference (Reese, 2005 ▸; Stelakatos *et al.*, 1959 ▸). In conclusion, the versatility of these ethers renders them indispensable tools in synthetic chemistry, enabling precise control over reactivity and facilitating the synthesis of complex mol­ecules. In the present work we report on the preparation conditions and crystal structures of 1,1′,1′′-[(2-bromo­eth­oxy)methane­tri­yl]tri­benzene (**1**) and 1,1′,1′′-[(2-iodo­eth­oxy)methane­tri­yl]tri­benzene (**2**).
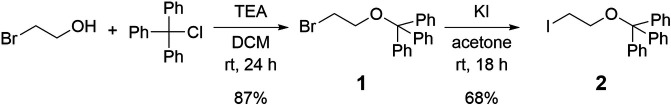


## 2. Structural commentary

**Figure 1:**
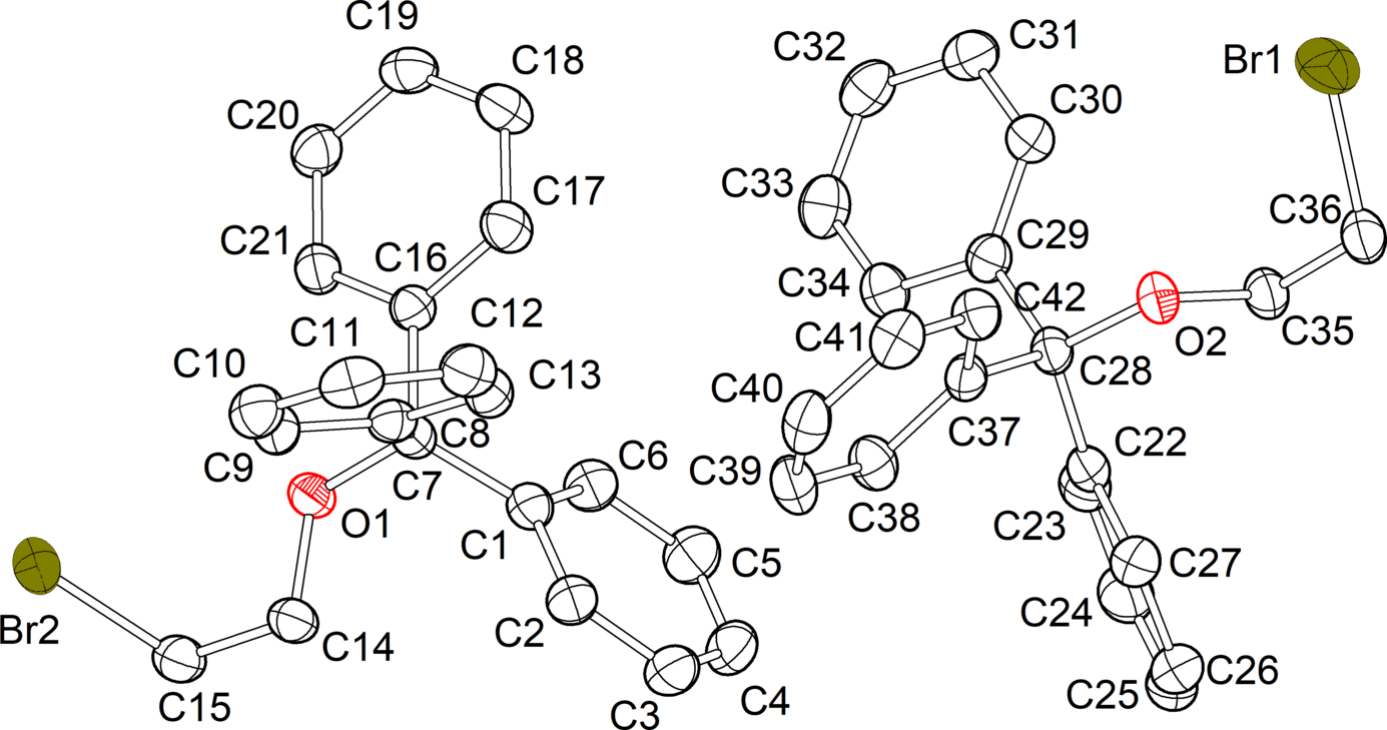
Asymmetric unit of the solid-state structure of compound **1** with the atom-labelling scheme. Displacement ellipsoids are shown at the 50% probability level and H atoms are omitted for clarity.

**Figure 2:**
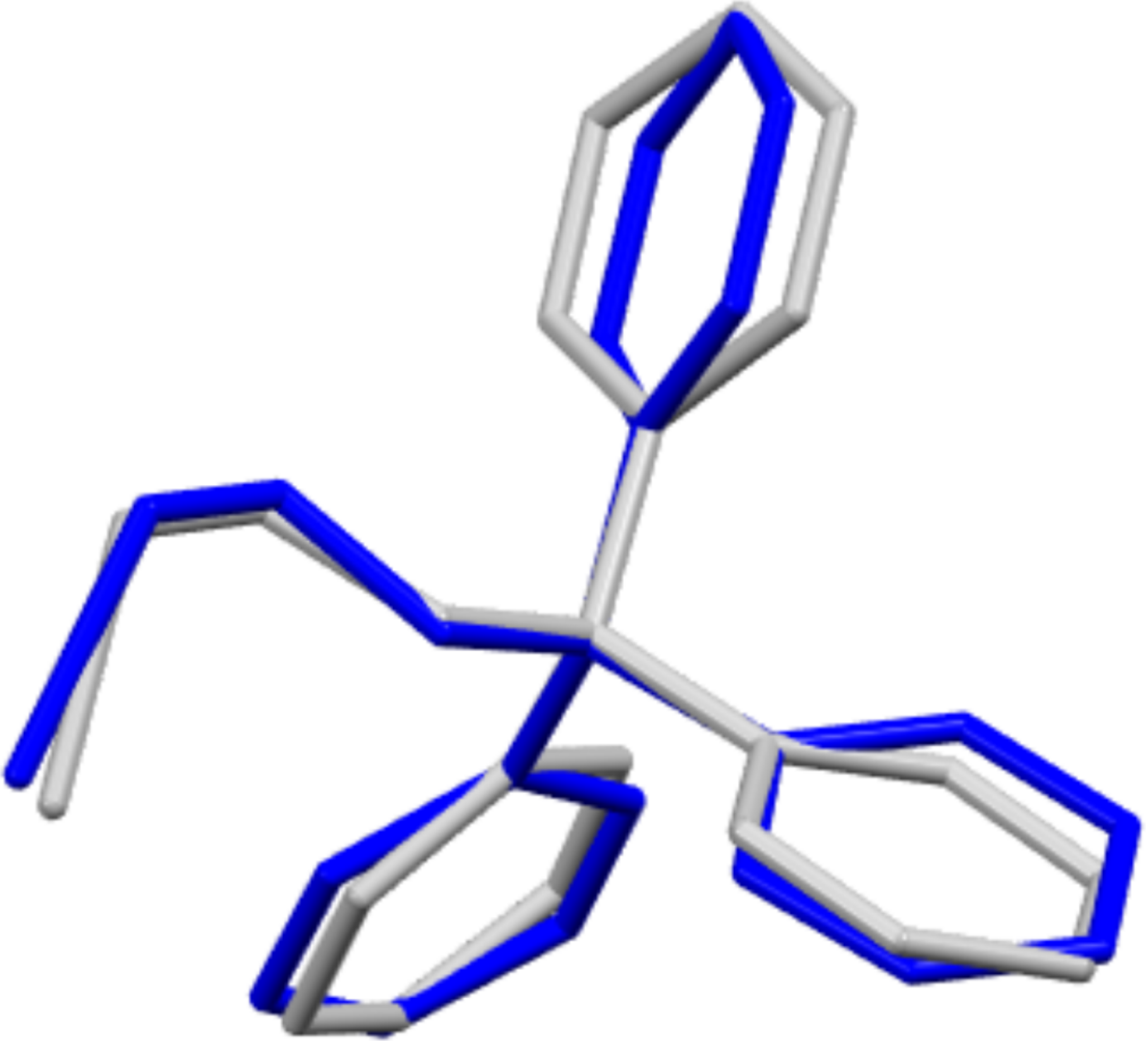
An overlay of the two independent mol­ecules in the asymmetric unit of the structure of compound **1** (r.m.s. deviation for non-hydrogen atoms: 0.284 Å).

**Figure 3:**
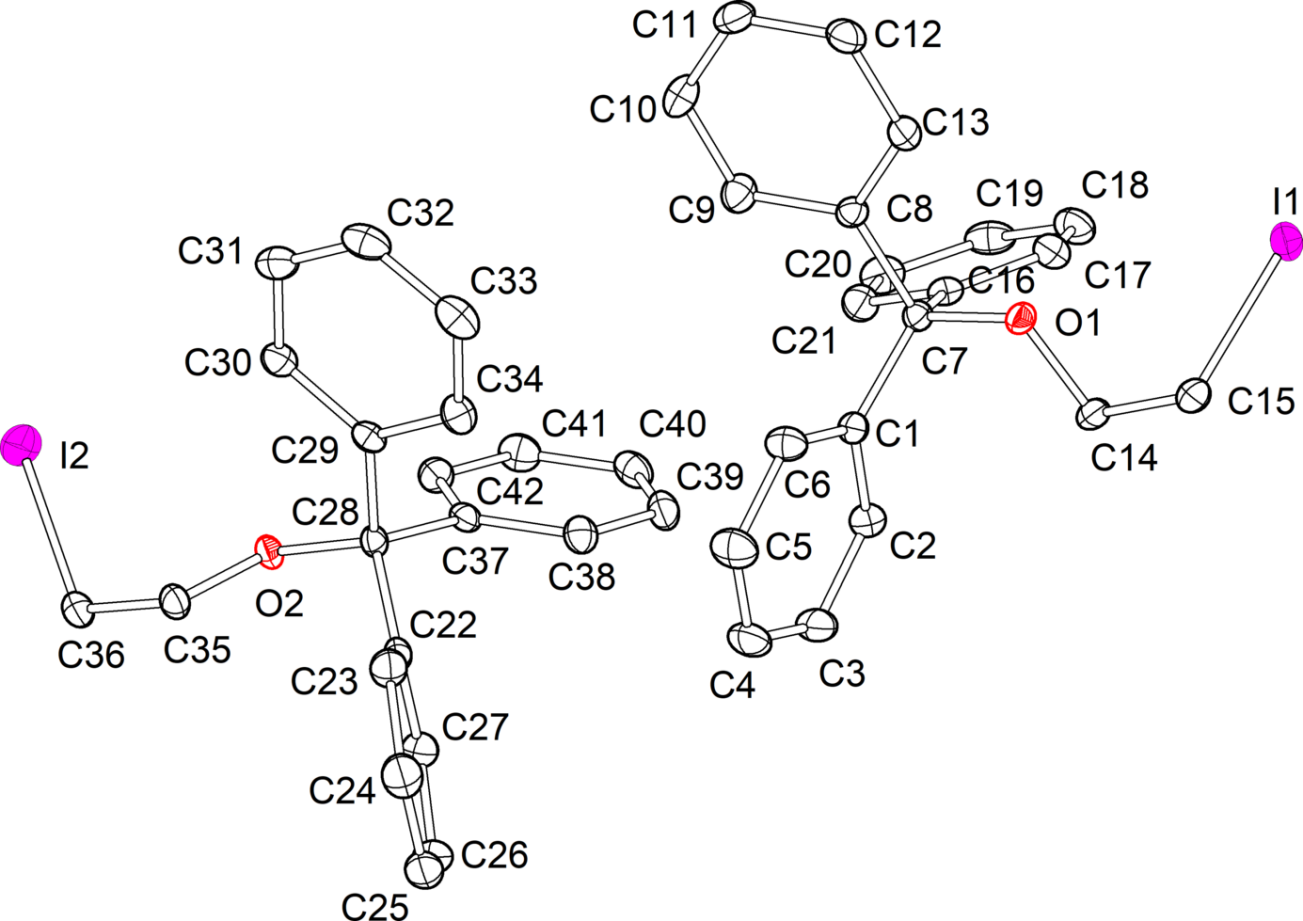
Asymmetric unit of the solid-state structure of compound **2** with the atom-labelling scheme. Displacement ellipsoids are shown at the 50% probability level and H atoms are omitted for clarity.

**Figure 4:**
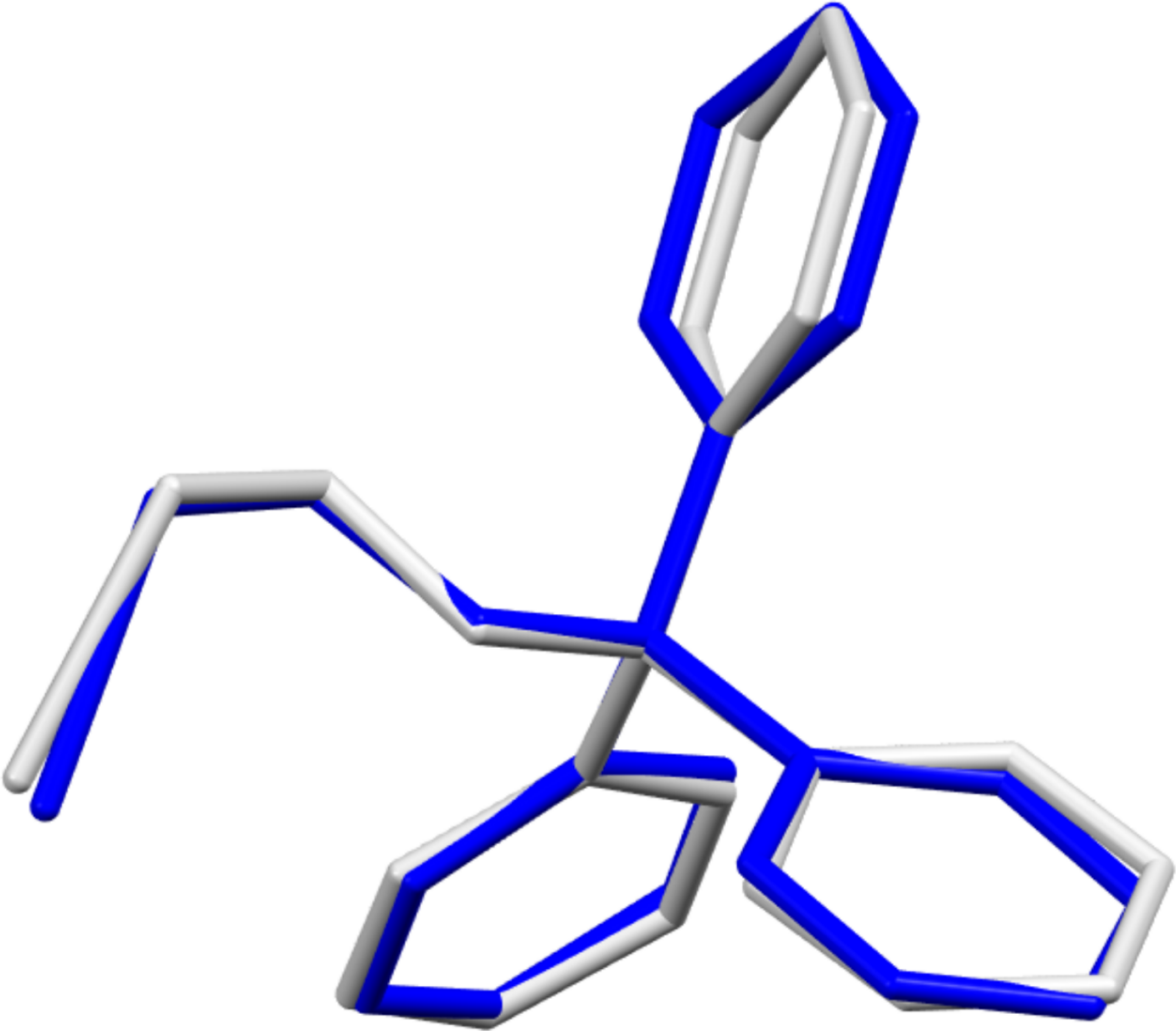
An overlay of the two independent mol­ecules in the asymmetric unit of the structure of compound **2** (r.m.s. deviation for non-hydrogen atoms: 0.181 Å).

Compound **1** crystallizes in the monoclinic space group *P*2_1_/*c* and comprises two independent mol­ecules in the asymmetric unit (Fig. 1 ▸). The C—Br distances of both mol­ecules are identical [1.960 (2) Å and 1.959 (2) Å], and fall within the range observed for comparable bromo­ethanol ether derivatives (1.921–1.942 Å, see *Database survey*). Nevertheless, the torsion angles of the alkyl chains (O-C-C-Br) display minor discrepancies [–65.16 (17) and −59.94 (17)°]. In general, the two mol­ecules of the asymmetric unit of compound **1** exhibit a comparable structural configuration, as evidenced by the superimposed structure (Fig. 2 ▸). The structural properties of compound **2** are analogous to those of compound **1**, with the mol­ecule crystallizing in the monoclinic space group *P*2_1_/*c* and comprising two independent mol­ecules in the asymmetric unit (Fig. 3 ▸). The C—I distances of both mol­ecules are identical [2.1555 (18) and 2.1533 (18) Å] and fall within the range of comparable iodo­ethanol ether derivatives (2.112–2.154 Å, see *Database survey*). Similarly, the torsion angles of the alkyl chains (O—C—C—I) exhibit slight differences, but comparable absolute values to those observed in compound **1**[−63.50 (15) and −58.47 (15)°]. However, the superimposed structure of the mol­ecules in the asymmetric unit of compound **2** (Fig. 4 ▸) illustrates a comparable structural configuration. No discernible intra­molecular inter­actions are evident in either compound **1** or compound **2**.

## 3. Supra­molecular features

**Figure 5:**
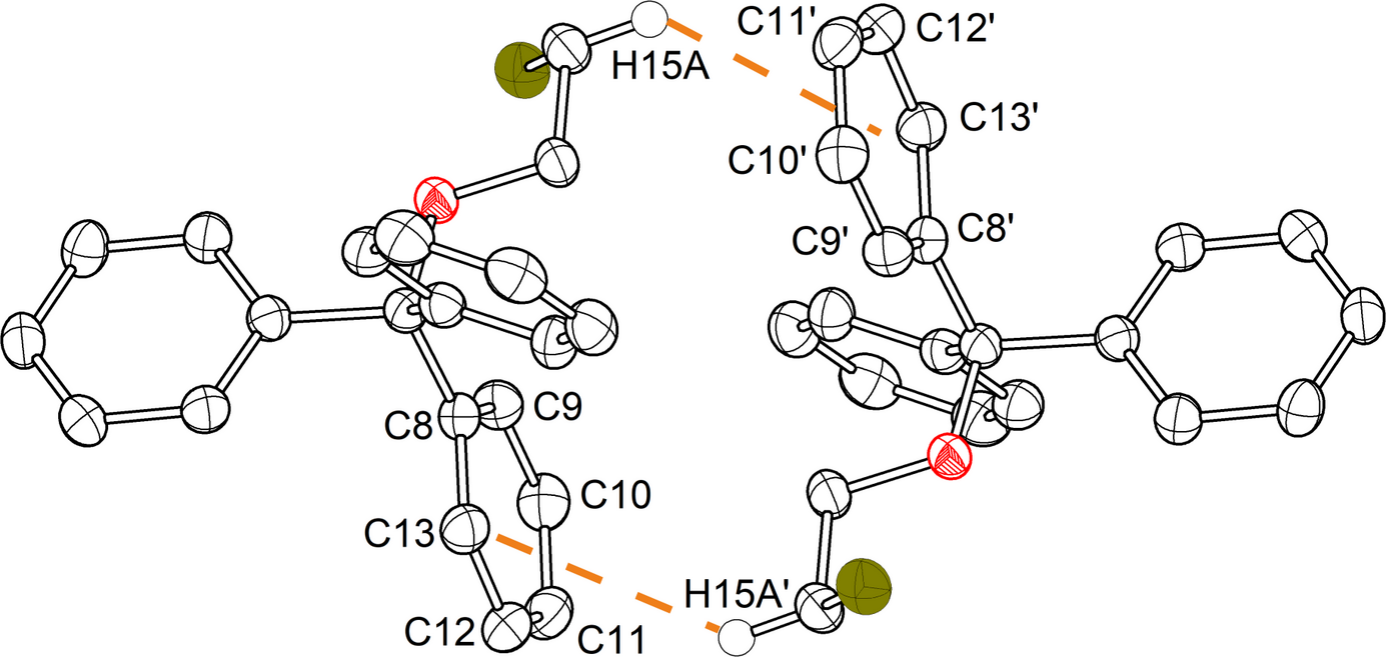
Partial packing diagram of compound **1** with highlighted C—H⋯π inter­actions of the proton H15*A* and the π-system of an adjacent phenyl ring of another mol­ecule. Displacement ellipsoids are shown at the 50% probability level, except for H15*A* and H15*A*′, which are shown at an arbitrary radius, and H atoms, except for H15*A* and H15*A*′, are omitted for clarity. Symmetry code: (’) 2 − *x*, −*y*, 1 − *z*.

The structural configuration of compound **2** does not exhibit any notable inter­molecular inter­actions. Conversely, one of the mol­ecules present within the asymmetric unit of compound **1** engages in inter­molecular inter­actions with another mol­ecule situated outside of the asymmetric unit. The mol­ecule forms a dimer-like structure (Fig. 5 ▸) with a symmetry-equivalent mol­ecule, in which a proton from the alkyl chain (H15*A*) has a very short contact to a phenyl ring (C8–C13) of the trityl unit of the other mol­ecule. The shortest distance is observed to the carbon atom C12 (2.78 Å). The distance of the hydrogen atom to the centroid of the aromatic compound (C8–C13) is 3.37 Å, or 2.69 Å to the ring plane. This distance to the ring plane thus falls within the typical range (2.75±0.10 Å) for C—H⋯π contacts of hydrogen atoms on *sp*^3^-hybridized carbon atoms and aromatic systems (Nishio, 2011 ▸).

## 4. Database survey

A search in the Cambridge Structural Database (CSD, Version 5.45, update June 2024; Groom *et al.*, 2016 ▸) for trityl-protected 2-halo­ethanol derivatives gave two hits (CSD refcodes ZANNOR and ZANNUX; Ho *et al.*, 1995 ▸). It should be noted, however, that these derivatives differ from the title compounds in that they are boronic acid ester derivatives. A database survey for halo­ethanol ether derivatives with an unsubstituted alkyl chain yielded five entries for bromo derivatives [CSD refcodes TASYOF (Wang *et al.*, 2022 ▸), KUPJIP (Farràs *et al.*, 2010 ▸), RANGOF (Karpus *et al.*, 2015 ▸), TOFJIH (Jakobsmeier *et al.*, 1996 ▸), XUHSAW (Gierszewski *et al.*, 2015 ▸)] and five entries for iodo derivatives [COZXIB (Wang *et al.*, 2019 ▸), KUPJOV (Farràs *et al.*, 2010 ▸), SUKFEL (Pruitt *et al.*, 2015 ▸), XODPAK (Zhang *et al.*, 2019 ▸), ZOFQIW (Cox *et al.*, 2014 ▸)]. The structural characteristics of the entries on the halo­ethanol ether derivatives with an unsubstituted alkyl chain were used in the discussion of the structural features of compounds **1** and **2**.

## 5. Synthesis and crystallization

**General Considerations:** All reagents were purchased from commercial suppliers and used without further purification. Di­chloro­methane was dried using calcium hydride and distilled before use. Reactions of the air-sensitive compounds were carried out under an inert argon atmosphere using the Schlenk line technique. NMR spectra were recorded on Bruker Avance(III) 400 and Avance(Neo) 400 instruments. NMR spectra were referenced to residual solvent peaks (CD_2_Cl_2_). Mass spectra were recorded on Bruker impact II. The single-crystal X-ray diffraction (SC-XRD) data were collected on Bruker D8 Venture diffractometer with Photon III CMOS detector with Mo *K*_α_ radiation (λ = 0.71073 Å) from a microfocus source (IμS).

**Synthesis overview**: Compound **1** was synthesized starting from bromo­ethanol. The alcohol group was protected under alkaline conditions using trityl chloride and the synthesis was conducted in accordance with the published conditions (Sureshan *et al.*, 2001 ▸). Trityl ether **1** was then converted to the iodinated derivative **2** in a Finkelstein reaction based on the reaction conditions of similar compounds published in the literature (Meguellati *et al.*, 2010 ▸). (TEA: tri­ethyl­amine; DCM: di­chloro­methane).

**Synthesis of compound 1**: 2-Bromo­ethanol (1.00 mL, 14.11 mmol) and anhydrous tri­ethyl­amine (5.60 mL) were dissolved in anhydrous di­chloro­methane (30 mL) and then chloro­tri­phenyl­methane (4.32 g, 15.49 mmol) was added. The mixture was stirred at room temperature for 16 h. A saturated solution of NaHCO_3_ (30 mL) was added, the mixture extracted with ethyl acetate (3 × 20 mL) and the combined organic phases were dried with MgSO_4_. The solvent was removed under reduced pressure and the crude product was purified by column chromatography (petroleum ether:ethyl acetate, 15:1 to 10:1, *R*_f_ = 0.9). Compound **1** was obtained as a white solid (5.17 g, 14.08 mmol, 99%). ^1^H NMR (400 MHz, CD_2_Cl_2_, 300 K, ppm): *δ* 7.51–7.45 (*m*, 6H), 7.37–7.30 (*m*, 6H), 7.30–7.25 (*m*, 3H), 1.46–1.42 (*m*, 4H). ^13^C{^1^H} NMR (100 MHz, CD_2_Cl_2_, 300 K, ppm): *δ* 129.01, 128.30, 127.58, 87.28, 64.23, 31.76. ESI-ESI-MS(+): *m*/*z* = 391.0513 ([*M*+Na]^+^, calculated: 391.0517 *m*/*z*). Single crystals of **1** suitable for X-ray diffraction were obtained by diffusion of *n*-hexane into a solution of compound **1** in THF at room temperature.

**Synthesis of compound 2**: Compound **1** (0.20 g, 0.54 mmol) was dissolved in acetone (20 mL) and potassium iodide (0.45 g, 2.72 mmol) was added. The suspension was refluxed at 338 K for 4 d and then the solution was filtered. Di­chloro­methane (50 mL) was added to the solution and the organic phase washed with water (2 × 20 mL). The organic phase was dried with MgSO_4_ and the solvent was removed under reduced pressure. Compound **2** was obtained as an off-white solid (0.15 g, 0.37 mmol, 69%). ^1^H NMR (400 MHz, CD_2_Cl_2_, 300 K, ppm): *δ* 7.48–7.45 (*m*, 6H), 7.35–7.30 (*m*, 6H), 7.29–7.24 (*m*, 3H), 3.38 (*t*, ^3^*J*_HH_= 6.5 Hz, 2H), 3.22–3.16 (*t*, ^3^*J*_HH_= 6.5 Hz, 2H). ^13^C{^1^H} NMR(100 MHz, CD_2_Cl_2_, 300 K, ppm): *δ* 144.39, 129.01, 128.29, 127.57, 87.32, 64.82, 4.20. ESI-MS(+): *m*/*z* = 437.0373 ([*M*+Na]^+^, calculated: 437.0378 *m*/*z*). Single crystals of **2** suitable for X-ray diffraction were obtained by diffusion of *n*-hexane into a solution of compound **2** in THF at room temperature.

## 6. Refinement

Crystal data, data collection and structure refinement details are summarized in Table 1 ▸. Hydrogen atoms were placed in ideal calculated positions and refined using a riding model.

## Supplementary Material

Crystal structure: contains datablock(s) 1, 2. DOI: 10.1107/S2056989025001124/yy2014sup1.cif

Structure factors: contains datablock(s) 1. DOI: 10.1107/S2056989025001124/yy20141sup2.hkl

Structure factors: contains datablock(s) 2. DOI: 10.1107/S2056989025001124/yy20142sup3.hkl

CCDC references: 2422019, 2422018

Additional supporting information:  crystallographic information; 3D view; checkCIF report

## Figures and Tables

**Table 1:** Experimental details

	**1**	**2**
Crystal data		
Chemical formula	C_21_H_19_BrO	C_21_H_19_IO
*M*_r_	367.27	414.26
Crystal system, space group	Monoclinic, *P*2_1_/*c*	Monoclinic, *P*2_1_/*c*
Temperature (K)	150	120
*a*, *b*, *c* (Å)	19.2056 (5), 10.5517 (3), 17.4704 (5)	19.3500 (6), 10.5936 (3), 17.5475 (6)
β (°)	107.512 (1)	105.945 (1)
*V* (Å^3^)	3376.32 (16)	3458.60 (19)
*Z*	8	8
Radiation type	Mo *K*α	Mo *K*α
μ (mm^−1^)	2.44	1.85
Crystal size (mm)	0.75 × 0.40 × 0.27	0.59 × 0.25 × 0.20

Data collection		
Diffractometer	Bruker D8 Venture with Photon III CMOS detector	Bruker D8 Venture with Photon III CMOS detector
Absorption correction	Empirical (using intensity measurements) (*SADABS*; Krause et al., 2015 ▸)	Empirical (using intensity measurements) (*SADABS*; Krause et al., 2015 ▸)
*T*_min_, *T*_max_	0.016, 0.050	0.506, 0.746
No. of measured, independent and observed [*I* > 2σ(*I*)] reflections	58221, 7760, 6794	47654, 7929, 7577
*R*_int_	0.041	0.028
(sin θ/λ)_max_ (Å^−1^)	0.650	0.650

Refinement		
*R*[*F*^2^ > 2σ(*F*^2^)], *wR*(*F*^2^), *S*	0.032, 0.088, 1.05	0.020, 0.054, 1.10
No. of reflections	7760	7929
No. of parameters	415	415
H-atom treatment	H-atom parameters constrained	H-atom parameters constrained
Δρ_max_, Δρ_min_ (e Å^−3^)	0.50, −0.70	0.56, −0.68

Computer programs: *APEX4* and *SAINT-Plus* (Bruker, 2021 ▸), *SHELXT2018/2* (Sheldrick, 2015*a* ▸), *SHELXL2019/1* (Sheldrick, 2015*b* ▸), *SHELXTL* (Sheldrick, 2008 ▸) and *publCIF* (Westrip, 2010 ▸).

## supplementary crystallographic information

### 1,1',1''-[(2-Bromoethoxy)methanetriyl]tribenzene (1) Crystal data

**Table:**

C_21_H_19_BrO	*F*(000) = 1504
*M**_r_* = 367.27	*D*_x_ = 1.445 Mg m^−^^3^
Monoclinic, *P*2_1_/*c*	Mo *K*α radiation, λ = 0.71073 Å
*a* = 19.2056 (5) Å	Cell parameters from 9414 reflections
*b* = 10.5517 (3) Å	θ = 2.2–27.5°
*c* = 17.4704 (5) Å	µ = 2.44 mm^−^^1^
β = 107.512 (1)°	*T* = 150 K
*V* = 3376.32 (16) Å^3^	Block, colourless
*Z* = 8	0.75 × 0.40 × 0.27 mm

### 1,1',1''-[(2-Bromoethoxy)methanetriyl]tribenzene (1) Data collection

**Table:**

Bruker D8 Venture with Photon III CMOS detector diffractometer	6794 reflections with *I* > 2σ(*I*)
Radiation source: microfocus source	*R*_int_ = 0.041
φ and ω scans	θ_max_ = 27.5°, θ_min_ = 2.2°
Absorption correction: empirical (using intensity measurements) (SADABS; Krause et al., 2015)	*h* = −24→24
*T*_min_ = 0.016, *T*_max_ = 0.050	*k* = −13→13
58221 measured reflections	*l* = −22→22
7760 independent reflections	

### 1,1',1''-[(2-Bromoethoxy)methanetriyl]tribenzene (1) Refinement

**Table:**

Refinement on *F*^2^	Primary atom site location: intrinsic phasing
Least-squares matrix: full	Hydrogen site location: inferred from neighbouring sites
*R*[*F*^2^ > 2σ(*F*^2^)] = 0.032	H-atom parameters constrained
*wR*(*F*^2^) = 0.088	*w* = 1/[σ^2^(*F*_o_^2^) + (0.0435*P*)^2^ + 1.5246*P*] where *P* = (*F*_o_^2^ + 2*F*_c_^2^)/3
*S* = 1.05	(Δ/σ)_max_ = 0.004
7760 reflections	Δρ_max_ = 0.50 e Å^−^^3^
415 parameters	Δρ_min_ = −0.70 e Å^−^^3^
0 restraints	

### 1,1',1''-[(2-Bromoethoxy)methanetriyl]tribenzene (1) Special details

**Table:**

Geometry. All esds (except the esd in the dihedral angle between two l.s. planes) are estimated using the full covariance matrix. The cell esds are taken into account individually in the estimation of esds in distances, angles and torsion angles; correlations between esds in cell parameters are only used when they are defined by crystal symmetry. An approximate (isotropic) treatment of cell esds is used for estimating esds involving l.s. planes.

### 1,1',1''-[(2-Bromoethoxy)methanetriyl]tribenzene (1) Fractional atomic coordinates and isotropic or equivalent isotropic displacement parameters (Å^2^)

**Table:**

	*x*	*y*	*z*	*U*_iso_*/*U*_eq_	
Br1	0.33236 (2)	0.28194 (2)	0.33521 (2)	0.04960 (8)	
Br2	1.16003 (2)	0.25401 (2)	0.44727 (2)	0.03928 (7)	
O1	1.00339 (7)	0.29832 (11)	0.46140 (7)	0.0279 (3)	
O2	0.49226 (7)	0.21218 (12)	0.44788 (7)	0.0272 (2)	
C1	0.89339 (10)	0.26796 (17)	0.49948 (11)	0.0284 (4)	
C2	0.88895 (10)	0.15444 (18)	0.53838 (11)	0.0331 (4)	
H2	0.902632	0.077618	0.518434	0.040*	
C3	0.86481 (11)	0.1514 (2)	0.60603 (12)	0.0394 (4)	
H3	0.862434	0.072997	0.631852	0.047*	
C4	0.84427 (12)	0.2624 (2)	0.63572 (12)	0.0405 (5)	
H4	0.827609	0.260454	0.681693	0.049*	
C5	0.84826 (12)	0.3758 (2)	0.59787 (12)	0.0400 (4)	
H5	0.833927	0.452190	0.617672	0.048*	
C6	0.87324 (10)	0.37888 (18)	0.53061 (11)	0.0333 (4)	
H6	0.876538	0.457757	0.505716	0.040*	
C7	0.92638 (9)	0.27394 (16)	0.42979 (11)	0.0265 (3)	
C8	0.91188 (10)	0.15403 (16)	0.37754 (10)	0.0273 (3)	
C9	0.96503 (10)	0.10559 (18)	0.34649 (11)	0.0316 (4)	
H9	1.011442	0.145521	0.359073	0.038*	
C10	0.95098 (11)	−0.00075 (19)	0.29718 (11)	0.0364 (4)	
H10	0.988467	−0.034632	0.278058	0.044*	
C11	0.88319 (12)	−0.05745 (18)	0.27579 (11)	0.0377 (4)	
H11	0.873863	−0.129718	0.241780	0.045*	
C12	0.82873 (11)	−0.00842 (18)	0.30417 (12)	0.0366 (4)	
H12	0.781482	−0.045555	0.288378	0.044*	
C13	0.84332 (10)	0.09562 (18)	0.35601 (11)	0.0324 (4)	
H13	0.806328	0.127013	0.376851	0.039*	
C14	1.04580 (10)	0.21427 (17)	0.52082 (11)	0.0304 (4)	
H14A	1.040800	0.126338	0.500110	0.036*	
H14B	1.029277	0.216889	0.569288	0.036*	
C15	1.12392 (11)	0.25704 (18)	0.54086 (12)	0.0341 (4)	
H15A	1.154853	0.201186	0.583053	0.041*	
H15B	1.128066	0.344286	0.562619	0.041*	
C16	0.89722 (9)	0.38741 (16)	0.37363 (10)	0.0261 (3)	
C17	0.82250 (10)	0.40218 (18)	0.33765 (11)	0.0336 (4)	
H17	0.789464	0.344306	0.349861	0.040*	
C18	0.79561 (11)	0.50073 (19)	0.28398 (12)	0.0368 (4)	
H18	0.744473	0.509288	0.259566	0.044*	
C19	0.84292 (11)	0.58624 (18)	0.26602 (11)	0.0344 (4)	
H19	0.824644	0.653005	0.228938	0.041*	
C20	0.91707 (11)	0.57351 (18)	0.30261 (11)	0.0347 (4)	
H20	0.949829	0.632918	0.291312	0.042*	
C21	0.94430 (10)	0.47447 (17)	0.35589 (11)	0.0309 (4)	
H21	0.995476	0.466436	0.380261	0.037*	
C22	0.59569 (10)	0.24168 (17)	0.56667 (11)	0.0282 (4)	
C23	0.62123 (10)	0.34940 (19)	0.61183 (11)	0.0340 (4)	
H23	0.625614	0.426583	0.585654	0.041*	
C24	0.64060 (12)	0.3453 (2)	0.69562 (12)	0.0426 (5)	
H24	0.658598	0.419384	0.726002	0.051*	
C25	0.63375 (12)	0.2345 (2)	0.73439 (13)	0.0457 (5)	
H25	0.646999	0.231914	0.791322	0.055*	
C26	0.60734 (12)	0.1265 (2)	0.68961 (12)	0.0434 (5)	
H26	0.601948	0.050045	0.715980	0.052*	
C27	0.58876 (10)	0.1299 (2)	0.60636 (11)	0.0357 (4)	
H27	0.571175	0.055374	0.576215	0.043*	
C28	0.56932 (9)	0.23937 (16)	0.47479 (10)	0.0255 (3)	
C29	0.58624 (10)	0.36120 (16)	0.43556 (10)	0.0267 (3)	
C30	0.53171 (10)	0.42653 (17)	0.37837 (10)	0.0304 (4)	
H30	0.482860	0.396409	0.363979	0.036*	
C31	0.54788 (11)	0.53525 (18)	0.34211 (11)	0.0348 (4)	
H31	0.509870	0.579766	0.304112	0.042*	
C32	0.61897 (12)	0.57911 (18)	0.36096 (11)	0.0362 (4)	
H32	0.629988	0.653306	0.336048	0.043*	
C33	0.67381 (11)	0.51347 (18)	0.41659 (12)	0.0373 (4)	
H33	0.722875	0.542085	0.429247	0.045*	
C34	0.65768 (10)	0.40633 (18)	0.45394 (11)	0.0326 (4)	
H34	0.695772	0.363043	0.492587	0.039*	
C35	0.44738 (10)	0.27958 (18)	0.48586 (11)	0.0304 (4)	
H35A	0.451085	0.371907	0.477773	0.036*	
H35B	0.463252	0.262141	0.544246	0.036*	
C36	0.37030 (10)	0.23595 (18)	0.44889 (12)	0.0343 (4)	
H36A	0.338883	0.274187	0.478418	0.041*	
H36B	0.368164	0.142733	0.454228	0.041*	
C37	0.60424 (9)	0.12823 (16)	0.44312 (10)	0.0262 (3)	
C38	0.67771 (10)	0.09807 (17)	0.47801 (12)	0.0322 (4)	
H38	0.706160	0.145549	0.522702	0.039*	
C39	0.70961 (10)	−0.00044 (17)	0.44813 (13)	0.0360 (4)	
H39	0.759860	−0.019286	0.471961	0.043*	
C40	0.66824 (11)	−0.07205 (17)	0.38322 (13)	0.0373 (4)	
H40	0.690075	−0.139979	0.363063	0.045*	
C41	0.59619 (11)	−0.04395 (19)	0.34883 (12)	0.0370 (4)	
H41	0.567694	−0.093270	0.305093	0.044*	
C42	0.56409 (10)	0.05703 (17)	0.37762 (11)	0.0311 (4)	
H42	0.514285	0.077300	0.352165	0.037*	

### 1,1',1''-[(2-Bromoethoxy)methanetriyl]tribenzene (1) Atomic displacement parameters (Å^2^)

**Table:**

	*U*^11^	*U*^22^	*U*^33^	*U*^12^	*U*^13^	*U*^23^
Br1	0.03491 (12)	0.05928 (15)	0.04485 (13)	−0.00747 (9)	−0.00275 (9)	0.00187 (10)
Br2	0.02986 (11)	0.04413 (12)	0.04559 (12)	0.00347 (8)	0.01397 (9)	0.00213 (8)
O1	0.0231 (6)	0.0277 (6)	0.0309 (6)	0.0003 (5)	0.0051 (5)	0.0044 (5)
O2	0.0213 (6)	0.0303 (6)	0.0309 (6)	−0.0005 (5)	0.0094 (5)	−0.0034 (5)
C1	0.0243 (8)	0.0308 (9)	0.0290 (8)	−0.0011 (7)	0.0063 (7)	0.0014 (7)
C2	0.0333 (10)	0.0317 (9)	0.0352 (9)	0.0015 (7)	0.0117 (8)	0.0056 (7)
C3	0.0377 (11)	0.0445 (11)	0.0357 (10)	−0.0026 (9)	0.0107 (8)	0.0097 (8)
C4	0.0374 (11)	0.0564 (13)	0.0290 (9)	−0.0030 (9)	0.0122 (8)	0.0006 (8)
C5	0.0439 (11)	0.0449 (11)	0.0323 (9)	0.0009 (9)	0.0130 (9)	−0.0066 (8)
C6	0.0359 (10)	0.0327 (9)	0.0311 (9)	−0.0010 (7)	0.0096 (8)	−0.0017 (7)
C7	0.0230 (8)	0.0258 (8)	0.0297 (8)	0.0006 (6)	0.0066 (7)	0.0027 (6)
C8	0.0295 (9)	0.0252 (8)	0.0263 (8)	0.0005 (7)	0.0071 (7)	0.0039 (6)
C9	0.0281 (9)	0.0336 (9)	0.0338 (9)	−0.0004 (7)	0.0105 (7)	0.0008 (7)
C10	0.0423 (11)	0.0355 (10)	0.0339 (9)	0.0047 (8)	0.0152 (8)	−0.0006 (8)
C11	0.0516 (12)	0.0280 (9)	0.0314 (9)	−0.0027 (8)	0.0091 (9)	−0.0010 (7)
C12	0.0359 (10)	0.0305 (9)	0.0410 (10)	−0.0053 (8)	0.0083 (8)	0.0039 (8)
C13	0.0293 (9)	0.0309 (9)	0.0374 (9)	−0.0016 (7)	0.0106 (8)	0.0030 (7)
C14	0.0271 (9)	0.0304 (9)	0.0313 (9)	0.0028 (7)	0.0053 (7)	0.0054 (7)
C15	0.0296 (10)	0.0368 (10)	0.0326 (9)	0.0006 (7)	0.0045 (8)	0.0050 (7)
C16	0.0262 (8)	0.0263 (8)	0.0260 (8)	0.0014 (6)	0.0080 (7)	0.0004 (6)
C17	0.0279 (9)	0.0309 (9)	0.0397 (10)	−0.0030 (7)	0.0069 (8)	0.0033 (8)
C18	0.0288 (9)	0.0361 (10)	0.0401 (10)	0.0042 (7)	0.0025 (8)	0.0045 (8)
C19	0.0418 (11)	0.0298 (9)	0.0305 (9)	0.0074 (8)	0.0090 (8)	0.0048 (7)
C20	0.0370 (10)	0.0304 (9)	0.0402 (10)	0.0016 (7)	0.0169 (8)	0.0066 (8)
C21	0.0266 (9)	0.0315 (9)	0.0360 (9)	0.0013 (7)	0.0114 (7)	0.0036 (7)
C22	0.0213 (8)	0.0356 (9)	0.0272 (8)	0.0022 (6)	0.0066 (7)	0.0011 (7)
C23	0.0320 (10)	0.0375 (10)	0.0320 (9)	0.0032 (8)	0.0086 (8)	−0.0027 (8)
C24	0.0356 (11)	0.0586 (13)	0.0311 (9)	0.0024 (9)	0.0064 (8)	−0.0091 (9)
C25	0.0318 (11)	0.0767 (16)	0.0275 (9)	0.0089 (10)	0.0073 (8)	0.0049 (10)
C26	0.0357 (11)	0.0578 (13)	0.0375 (10)	0.0031 (9)	0.0122 (9)	0.0152 (9)
C27	0.0318 (10)	0.0413 (10)	0.0344 (9)	−0.0005 (8)	0.0106 (8)	0.0052 (8)
C28	0.0210 (8)	0.0288 (8)	0.0270 (8)	−0.0010 (6)	0.0076 (7)	−0.0014 (6)
C29	0.0283 (9)	0.0264 (8)	0.0267 (8)	0.0002 (7)	0.0104 (7)	−0.0018 (6)
C30	0.0283 (9)	0.0336 (9)	0.0290 (8)	0.0009 (7)	0.0082 (7)	0.0008 (7)
C31	0.0425 (11)	0.0326 (9)	0.0301 (9)	0.0035 (8)	0.0122 (8)	0.0017 (7)
C32	0.0499 (12)	0.0269 (9)	0.0371 (10)	−0.0031 (8)	0.0212 (9)	−0.0007 (7)
C33	0.0369 (10)	0.0346 (10)	0.0435 (11)	−0.0091 (8)	0.0166 (9)	−0.0073 (8)
C34	0.0279 (9)	0.0321 (9)	0.0375 (9)	−0.0019 (7)	0.0097 (8)	−0.0031 (7)
C35	0.0253 (9)	0.0352 (9)	0.0315 (9)	0.0027 (7)	0.0100 (7)	−0.0017 (7)
C36	0.0248 (9)	0.0384 (10)	0.0413 (10)	0.0011 (7)	0.0124 (8)	0.0028 (8)
C37	0.0242 (8)	0.0273 (8)	0.0293 (8)	−0.0013 (6)	0.0114 (7)	0.0012 (6)
C38	0.0269 (9)	0.0293 (9)	0.0398 (10)	−0.0021 (7)	0.0093 (8)	−0.0015 (7)
C39	0.0268 (9)	0.0294 (9)	0.0542 (12)	0.0016 (7)	0.0158 (9)	0.0041 (8)
C40	0.0450 (11)	0.0242 (8)	0.0525 (11)	0.0026 (8)	0.0294 (10)	0.0002 (8)
C41	0.0425 (11)	0.0338 (10)	0.0373 (10)	−0.0057 (8)	0.0158 (9)	−0.0053 (8)
C42	0.0294 (9)	0.0319 (9)	0.0323 (9)	−0.0006 (7)	0.0094 (7)	−0.0013 (7)

### 1,1',1''-[(2-Bromoethoxy)methanetriyl]tribenzene (1) Geometric parameters (Å, º)

**Table:**

Br1—C36	1.960 (2)	C20—C21	1.393 (2)
Br2—C15	1.959 (2)	C20—H20	0.9500
O1—C14	1.421 (2)	C21—H21	0.9500
O1—C7	1.438 (2)	C22—C23	1.386 (3)
O2—C35	1.426 (2)	C22—C27	1.395 (3)
O2—C28	1.441 (2)	C22—C28	1.531 (2)
C1—C2	1.392 (2)	C23—C24	1.398 (3)
C1—C6	1.394 (3)	C23—H23	0.9500
C1—C7	1.534 (3)	C24—C25	1.377 (3)
C2—C3	1.394 (3)	C24—H24	0.9500
C2—H2	0.9500	C25—C26	1.389 (3)
C3—C4	1.385 (3)	C25—H25	0.9500
C3—H3	0.9500	C26—C27	1.390 (3)
C4—C5	1.381 (3)	C26—H26	0.9500
C4—H4	0.9500	C27—H27	0.9500
C5—C6	1.397 (3)	C28—C37	1.534 (2)
C5—H5	0.9500	C28—C29	1.537 (2)
C6—H6	0.9500	C29—C30	1.393 (2)
C7—C8	1.536 (2)	C29—C34	1.395 (2)
C7—C16	1.543 (2)	C30—C31	1.390 (3)
C8—C9	1.390 (2)	C30—H30	0.9500
C8—C13	1.399 (2)	C31—C32	1.384 (3)
C9—C10	1.391 (3)	C31—H31	0.9500
C9—H9	0.9500	C32—C33	1.385 (3)
C10—C11	1.378 (3)	C32—H32	0.9500
C10—H10	0.9500	C33—C34	1.386 (3)
C11—C12	1.385 (3)	C33—H33	0.9500
C11—H11	0.9500	C34—H34	0.9500
C12—C13	1.397 (3)	C35—C36	1.498 (3)
C12—H12	0.9500	C35—H35A	0.9900
C13—H13	0.9500	C35—H35B	0.9900
C14—C15	1.504 (3)	C36—H36A	0.9900
C14—H14A	0.9900	C36—H36B	0.9900
C14—H14B	0.9900	C37—C42	1.392 (2)
C15—H15A	0.9900	C37—C38	1.395 (2)
C15—H15B	0.9900	C38—C39	1.386 (3)
C16—C21	1.388 (2)	C38—H38	0.9500
C16—C17	1.391 (2)	C39—C40	1.395 (3)
C17—C18	1.391 (3)	C39—H39	0.9500
C17—H17	0.9500	C40—C41	1.365 (3)
C18—C19	1.382 (3)	C40—H40	0.9500
C18—H18	0.9500	C41—C42	1.398 (3)
C19—C20	1.381 (3)	C41—H41	0.9500
C19—H19	0.9500	C42—H42	0.9500
			
C14—O1—C7	117.79 (13)	C20—C21—H21	119.8
C35—O2—C28	117.02 (13)	C23—C22—C27	118.75 (17)
C2—C1—C6	117.85 (17)	C23—C22—C28	123.94 (16)
C2—C1—C7	121.56 (16)	C27—C22—C28	117.18 (16)
C6—C1—C7	120.32 (16)	C22—C23—C24	120.5 (2)
C1—C2—C3	121.23 (19)	C22—C23—H23	119.8
C1—C2—H2	119.4	C24—C23—H23	119.8
C3—C2—H2	119.4	C25—C24—C23	120.4 (2)
C4—C3—C2	120.20 (19)	C25—C24—H24	119.8
C4—C3—H3	119.9	C23—C24—H24	119.8
C2—C3—H3	119.9	C24—C25—C26	119.48 (19)
C5—C4—C3	119.36 (19)	C24—C25—H25	120.3
C5—C4—H4	120.3	C26—C25—H25	120.3
C3—C4—H4	120.3	C25—C26—C27	120.2 (2)
C4—C5—C6	120.38 (19)	C25—C26—H26	119.9
C4—C5—H5	119.8	C27—C26—H26	119.9
C6—C5—H5	119.8	C26—C27—C22	120.6 (2)
C1—C6—C5	120.98 (18)	C26—C27—H27	119.7
C1—C6—H6	119.5	C22—C27—H27	119.7
C5—C6—H6	119.5	O2—C28—C22	109.18 (14)
O1—C7—C1	108.99 (14)	O2—C28—C37	104.34 (13)
O1—C7—C8	110.93 (14)	C22—C28—C37	110.34 (14)
C1—C7—C8	113.18 (14)	O2—C28—C29	110.82 (14)
O1—C7—C16	103.89 (13)	C22—C28—C29	113.99 (14)
C1—C7—C16	112.29 (14)	C37—C28—C29	107.73 (14)
C8—C7—C16	107.17 (14)	C30—C29—C34	118.11 (17)
C9—C8—C13	118.40 (17)	C30—C29—C28	121.30 (16)
C9—C8—C7	120.79 (16)	C34—C29—C28	120.53 (16)
C13—C8—C7	120.69 (16)	C31—C30—C29	120.82 (18)
C8—C9—C10	120.68 (18)	C31—C30—H30	119.6
C8—C9—H9	119.7	C29—C30—H30	119.6
C10—C9—H9	119.7	C32—C31—C30	120.47 (18)
C11—C10—C9	120.63 (18)	C32—C31—H31	119.8
C11—C10—H10	119.7	C30—C31—H31	119.8
C9—C10—H10	119.7	C31—C32—C33	119.16 (17)
C10—C11—C12	119.61 (18)	C31—C32—H32	120.4
C10—C11—H11	120.2	C33—C32—H32	120.4
C12—C11—H11	120.2	C32—C33—C34	120.51 (18)
C11—C12—C13	119.99 (18)	C32—C33—H33	119.7
C11—C12—H12	120.0	C34—C33—H33	119.7
C13—C12—H12	120.0	C33—C34—C29	120.90 (18)
C12—C13—C8	120.62 (18)	C33—C34—H34	119.5
C12—C13—H13	119.7	C29—C34—H34	119.5
C8—C13—H13	119.7	O2—C35—C36	107.66 (15)
O1—C14—C15	107.17 (14)	O2—C35—H35A	110.2
O1—C14—H14A	110.3	C36—C35—H35A	110.2
C15—C14—H14A	110.3	O2—C35—H35B	110.2
O1—C14—H14B	110.3	C36—C35—H35B	110.2
C15—C14—H14B	110.3	H35A—C35—H35B	108.5
H14A—C14—H14B	108.5	C35—C36—Br1	112.24 (13)
C14—C15—Br2	112.32 (13)	C35—C36—H36A	109.2
C14—C15—H15A	109.1	Br1—C36—H36A	109.2
Br2—C15—H15A	109.1	C35—C36—H36B	109.2
C14—C15—H15B	109.1	Br1—C36—H36B	109.2
Br2—C15—H15B	109.1	H36A—C36—H36B	107.9
H15A—C15—H15B	107.9	C42—C37—C38	118.25 (16)
C21—C16—C17	118.54 (16)	C42—C37—C28	120.99 (15)
C21—C16—C7	121.30 (15)	C38—C37—C28	120.74 (15)
C17—C16—C7	120.14 (15)	C39—C38—C37	120.68 (18)
C18—C17—C16	120.76 (17)	C39—C38—H38	119.7
C18—C17—H17	119.6	C37—C38—H38	119.7
C16—C17—H17	119.6	C38—C39—C40	120.28 (18)
C19—C18—C17	120.32 (18)	C38—C39—H39	119.9
C19—C18—H18	119.8	C40—C39—H39	119.9
C17—C18—H18	119.8	C41—C40—C39	119.61 (17)
C20—C19—C18	119.24 (17)	C41—C40—H40	120.2
C20—C19—H19	120.4	C39—C40—H40	120.2
C18—C19—H19	120.4	C40—C41—C42	120.36 (18)
C19—C20—C21	120.67 (18)	C40—C41—H41	119.8
C19—C20—H20	119.7	C42—C41—H41	119.8
C21—C20—H20	119.7	C37—C42—C41	120.79 (17)
C16—C21—C20	120.47 (17)	C37—C42—H42	119.6
C16—C21—H21	119.8	C41—C42—H42	119.6
			
C6—C1—C2—C3	0.3 (3)	C27—C22—C23—C24	0.9 (3)
C7—C1—C2—C3	174.35 (18)	C28—C22—C23—C24	176.48 (18)
C1—C2—C3—C4	0.3 (3)	C22—C23—C24—C25	−0.8 (3)
C2—C3—C4—C5	−0.3 (3)	C23—C24—C25—C26	−0.1 (3)
C3—C4—C5—C6	−0.5 (3)	C24—C25—C26—C27	0.7 (3)
C2—C1—C6—C5	−1.0 (3)	C25—C26—C27—C22	−0.6 (3)
C7—C1—C6—C5	−175.15 (17)	C23—C22—C27—C26	−0.2 (3)
C4—C5—C6—C1	1.1 (3)	C28—C22—C27—C26	−176.10 (18)
C14—O1—C7—C1	55.44 (19)	C35—O2—C28—C22	45.52 (19)
C14—O1—C7—C8	−69.83 (18)	C35—O2—C28—C37	163.48 (14)
C14—O1—C7—C16	175.32 (14)	C35—O2—C28—C29	−80.84 (17)
C2—C1—C7—O1	−90.2 (2)	C23—C22—C28—O2	−113.98 (19)
C6—C1—C7—O1	83.7 (2)	C27—C22—C28—O2	61.7 (2)
C2—C1—C7—C8	33.7 (2)	C23—C22—C28—C37	131.90 (18)
C6—C1—C7—C8	−152.38 (16)	C27—C22—C28—C37	−52.4 (2)
C2—C1—C7—C16	155.23 (17)	C23—C22—C28—C29	10.5 (2)
C6—C1—C7—C16	−30.9 (2)	C27—C22—C28—C29	−173.80 (16)
O1—C7—C8—C9	−18.9 (2)	O2—C28—C29—C30	−3.6 (2)
C1—C7—C8—C9	−141.83 (16)	C22—C28—C29—C30	−127.28 (17)
C16—C7—C8—C9	93.83 (19)	C37—C28—C29—C30	109.92 (18)
O1—C7—C8—C13	165.05 (15)	O2—C28—C29—C34	179.16 (15)
C1—C7—C8—C13	42.2 (2)	C22—C28—C29—C34	55.5 (2)
C16—C7—C8—C13	−82.17 (19)	C37—C28—C29—C34	−67.3 (2)
C13—C8—C9—C10	−1.9 (3)	C34—C29—C30—C31	−1.5 (3)
C7—C8—C9—C10	−178.02 (16)	C28—C29—C30—C31	−178.72 (16)
C8—C9—C10—C11	2.4 (3)	C29—C30—C31—C32	1.4 (3)
C9—C10—C11—C12	−0.4 (3)	C30—C31—C32—C33	−0.2 (3)
C10—C11—C12—C13	−1.8 (3)	C31—C32—C33—C34	−1.0 (3)
C11—C12—C13—C8	2.3 (3)	C32—C33—C34—C29	1.0 (3)
C9—C8—C13—C12	−0.4 (3)	C30—C29—C34—C33	0.3 (3)
C7—C8—C13—C12	175.74 (16)	C28—C29—C34—C33	177.56 (17)
C7—O1—C14—C15	177.63 (15)	C28—O2—C35—C36	−179.74 (14)
O1—C14—C15—Br2	−59.94 (17)	O2—C35—C36—Br1	−65.16 (17)
O1—C7—C16—C21	8.0 (2)	O2—C28—C37—C42	23.1 (2)
C1—C7—C16—C21	125.59 (18)	C22—C28—C37—C42	140.26 (16)
C8—C7—C16—C21	−109.53 (18)	C29—C28—C37—C42	−94.72 (18)
O1—C7—C16—C17	−173.98 (15)	O2—C28—C37—C38	−158.60 (15)
C1—C7—C16—C17	−56.4 (2)	C22—C28—C37—C38	−41.5 (2)
C8—C7—C16—C17	68.5 (2)	C29—C28—C37—C38	83.57 (19)
C21—C16—C17—C18	1.1 (3)	C42—C37—C38—C39	−0.1 (3)
C7—C16—C17—C18	−177.01 (17)	C28—C37—C38—C39	−178.46 (17)
C16—C17—C18—C19	−0.5 (3)	C37—C38—C39—C40	−0.9 (3)
C17—C18—C19—C20	−0.7 (3)	C38—C39—C40—C41	0.5 (3)
C18—C19—C20—C21	1.2 (3)	C39—C40—C41—C42	0.9 (3)
C17—C16—C21—C20	−0.6 (3)	C38—C37—C42—C41	1.5 (3)
C7—C16—C21—C20	177.49 (16)	C28—C37—C42—C41	179.83 (16)
C19—C20—C21—C16	−0.6 (3)	C40—C41—C42—C37	−1.9 (3)

### 1,1',1''-[(2-Iodoethoxy)methanetriyl]tribenzene (2) Crystal data

**Table:**

C_21_H_19_IO	*F*(000) = 1648
*M**_r_* = 414.26	*D*_x_ = 1.591 Mg m^−^^3^
Monoclinic, *P*2_1_/*c*	Mo *K*α radiation, λ = 0.71073 Å
*a* = 19.3500 (6) Å	Cell parameters from 9904 reflections
*b* = 10.5936 (3) Å	θ = 2.2–27.5°
*c* = 17.5475 (6) Å	µ = 1.85 mm^−^^1^
β = 105.945 (1)°	*T* = 120 K
*V* = 3458.60 (19) Å^3^	Block, colourless
*Z* = 8	0.59 × 0.25 × 0.20 mm

### 1,1',1''-[(2-Iodoethoxy)methanetriyl]tribenzene (2) Data collection

**Table:**

Bruker D8 Venture with Photon III CMOS detector diffractometer	7577 reflections with *I* > 2σ(*I*)
Radiation source: microfocus source	*R*_int_ = 0.028
φ and ω scans	θ_max_ = 27.5°, θ_min_ = 2.2°
Absorption correction: empirical (using intensity measurements) (SADABS; Krause et al., 2015)	*h* = −25→25
*T*_min_ = 0.506, *T*_max_ = 0.746	*k* = −13→13
47654 measured reflections	*l* = −22→22
7929 independent reflections	

### 1,1',1''-[(2-Iodoethoxy)methanetriyl]tribenzene (2) Refinement

**Table:**

Refinement on *F*^2^	Primary atom site location: intrinsic phasing
Least-squares matrix: full	Hydrogen site location: inferred from neighbouring sites
*R*[*F*^2^ > 2σ(*F*^2^)] = 0.020	H-atom parameters constrained
*wR*(*F*^2^) = 0.054	*w* = 1/[σ^2^(*F*_o_^2^) + (0.0207*P*)^2^ + 2.7021*P*] where *P* = (*F*_o_^2^ + 2*F*_c_^2^)/3
*S* = 1.10	(Δ/σ)_max_ = 0.010
7929 reflections	Δρ_max_ = 0.56 e Å^−^^3^
415 parameters	Δρ_min_ = −0.68 e Å^−^^3^
0 restraints	

### 1,1',1''-[(2-Iodoethoxy)methanetriyl]tribenzene (2) Special details

**Table:**

Geometry. All esds (except the esd in the dihedral angle between two l.s. planes) are estimated using the full covariance matrix. The cell esds are taken into account individually in the estimation of esds in distances, angles and torsion angles; correlations between esds in cell parameters are only used when they are defined by crystal symmetry. An approximate (isotropic) treatment of cell esds is used for estimating esds involving l.s. planes.

### 1,1',1''-[(2-Iodoethoxy)methanetriyl]tribenzene (2) Fractional atomic coordinates and isotropic or equivalent isotropic displacement parameters (Å^2^)

**Table:**

	*x*	*y*	*z*	*U*_iso_*/*U*_eq_	
I1	1.16435 (2)	0.25441 (2)	0.44732 (2)	0.02017 (4)	
I2	0.32992 (2)	0.27263 (2)	0.33986 (2)	0.02538 (4)	
O1	1.00147 (6)	0.29359 (11)	0.46228 (7)	0.0143 (2)	
O2	0.49458 (6)	0.21182 (11)	0.45316 (6)	0.0135 (2)	
C1	0.89117 (9)	0.26274 (15)	0.49969 (10)	0.0146 (3)	
C2	0.88197 (9)	0.14767 (16)	0.53452 (10)	0.0184 (3)	
H2	0.892967	0.071211	0.512081	0.022*	
C3	0.85693 (10)	0.14342 (18)	0.60166 (10)	0.0226 (4)	
H3	0.851344	0.064306	0.624755	0.027*	
C4	0.84011 (11)	0.25365 (18)	0.63498 (11)	0.0250 (4)	
H4	0.822719	0.250573	0.680583	0.030*	
C5	0.84896 (11)	0.36882 (18)	0.60098 (11)	0.0257 (4)	
H5	0.837354	0.444935	0.623315	0.031*	
C6	0.87475 (10)	0.37331 (17)	0.53432 (10)	0.0198 (3)	
H6	0.881254	0.452728	0.512114	0.024*	
C7	0.92551 (9)	0.27031 (14)	0.43093 (10)	0.0130 (3)	
C8	0.89840 (8)	0.38360 (15)	0.37612 (9)	0.0139 (3)	
C9	0.82481 (9)	0.40409 (16)	0.34476 (10)	0.0193 (3)	
H9	0.791488	0.350778	0.360098	0.023*	
C10	0.79970 (9)	0.50165 (17)	0.29133 (10)	0.0214 (3)	
H10	0.749457	0.513496	0.269666	0.026*	
C11	0.84765 (10)	0.58170 (16)	0.26953 (10)	0.0206 (3)	
H11	0.830508	0.648466	0.233091	0.025*	
C12	0.92086 (10)	0.56340 (17)	0.30142 (10)	0.0212 (3)	
H12	0.954070	0.618438	0.287244	0.025*	
C13	0.94595 (9)	0.46448 (16)	0.35423 (10)	0.0188 (3)	
H13	0.996228	0.452374	0.375454	0.023*	
C14	1.04082 (9)	0.21454 (16)	0.52401 (10)	0.0168 (3)	
H14A	1.037747	0.125618	0.506024	0.020*	
H14B	1.021282	0.220309	0.570445	0.020*	
C15	1.11793 (9)	0.25909 (17)	0.54577 (10)	0.0192 (3)	
H15A	1.146904	0.205103	0.588853	0.023*	
H15B	1.120137	0.346554	0.566179	0.023*	
C16	0.91233 (9)	0.15102 (15)	0.37874 (9)	0.0146 (3)	
C17	0.96702 (9)	0.09650 (16)	0.35284 (10)	0.0184 (3)	
H17	1.013970	0.131661	0.368737	0.022*	
C18	0.95380 (10)	−0.00968 (17)	0.30358 (10)	0.0215 (3)	
H18	0.992059	−0.047714	0.287641	0.026*	
C19	0.88532 (10)	−0.05946 (17)	0.27802 (10)	0.0226 (4)	
H19	0.876336	−0.131444	0.244452	0.027*	
C20	0.82975 (10)	−0.00354 (17)	0.30175 (10)	0.0224 (4)	
H20	0.782317	−0.036027	0.283206	0.027*	
C21	0.84316 (9)	0.09963 (16)	0.35245 (10)	0.0188 (3)	
H21	0.805015	0.135769	0.369509	0.023*	
C22	0.59861 (9)	0.23780 (15)	0.56595 (10)	0.0148 (3)	
C23	0.62049 (9)	0.34627 (17)	0.61073 (10)	0.0196 (3)	
H23	0.622561	0.424361	0.584830	0.024*	
C24	0.63943 (10)	0.3414 (2)	0.69344 (11)	0.0261 (4)	
H24	0.654785	0.415979	0.723407	0.031*	
C25	0.63596 (10)	0.2287 (2)	0.73188 (11)	0.0271 (4)	
H25	0.648746	0.225647	0.788135	0.033*	
C26	0.61367 (10)	0.12002 (19)	0.68781 (11)	0.0253 (4)	
H26	0.611083	0.042351	0.713950	0.030*	
C27	0.59519 (9)	0.12458 (17)	0.60578 (10)	0.0199 (3)	
H27	0.579973	0.049688	0.576142	0.024*	
C28	0.57091 (8)	0.23694 (14)	0.47546 (10)	0.0127 (3)	
C29	0.58691 (9)	0.35892 (15)	0.43583 (9)	0.0142 (3)	
C30	0.53330 (9)	0.42041 (16)	0.37868 (10)	0.0172 (3)	
H30	0.485593	0.388467	0.365112	0.021*	
C31	0.54885 (10)	0.52858 (16)	0.34105 (10)	0.0207 (3)	
H31	0.511542	0.570912	0.303042	0.025*	
C32	0.61843 (10)	0.57428 (16)	0.35894 (11)	0.0229 (4)	
H32	0.629099	0.647588	0.333112	0.027*	
C33	0.67259 (10)	0.51230 (17)	0.41492 (11)	0.0224 (4)	
H33	0.720532	0.542899	0.426990	0.027*	
C34	0.65709 (9)	0.40608 (16)	0.45326 (10)	0.0187 (3)	
H34	0.694475	0.364833	0.491776	0.022*	
C35	0.45254 (9)	0.28117 (16)	0.49365 (10)	0.0161 (3)	
H35A	0.455105	0.372560	0.482955	0.019*	
H35B	0.470727	0.267344	0.551521	0.019*	
C36	0.37629 (9)	0.23547 (16)	0.46408 (10)	0.0180 (3)	
H36A	0.346791	0.277277	0.494738	0.022*	
H36B	0.375041	0.143462	0.473478	0.022*	
C37	0.60309 (8)	0.12667 (15)	0.44019 (9)	0.0137 (3)	
C38	0.67628 (9)	0.09875 (16)	0.46852 (10)	0.0175 (3)	
H38	0.705761	0.146919	0.510740	0.021*	
C39	0.70616 (9)	0.00127 (16)	0.43544 (11)	0.0194 (3)	
H39	0.756081	−0.016259	0.454777	0.023*	
C40	0.66371 (10)	−0.07091 (16)	0.37429 (11)	0.0204 (3)	
H40	0.684340	−0.137889	0.351947	0.024*	
C41	0.59110 (10)	−0.04451 (16)	0.34613 (10)	0.0207 (3)	
H41	0.561597	−0.094281	0.304789	0.025*	
C42	0.56118 (9)	0.05473 (16)	0.37821 (10)	0.0175 (3)	
H42	0.511548	0.073612	0.357549	0.021*	

### 1,1',1''-[(2-Iodoethoxy)methanetriyl]tribenzene (2) Atomic displacement parameters (Å^2^)

**Table:**

	*U*^11^	*U*^22^	*U*^33^	*U*^12^	*U*^13^	*U*^23^
I1	0.01599 (6)	0.02242 (6)	0.02299 (7)	0.00106 (4)	0.00688 (5)	0.00018 (4)
I2	0.01860 (6)	0.03365 (7)	0.02017 (6)	−0.00402 (5)	−0.00091 (5)	−0.00040 (5)
O1	0.0110 (5)	0.0158 (5)	0.0151 (5)	−0.0004 (4)	0.0017 (4)	0.0032 (4)
O2	0.0101 (5)	0.0170 (5)	0.0140 (5)	−0.0006 (4)	0.0043 (4)	−0.0024 (4)
C1	0.0117 (7)	0.0179 (8)	0.0138 (7)	−0.0003 (6)	0.0025 (6)	0.0011 (6)
C2	0.0194 (8)	0.0183 (8)	0.0178 (8)	0.0009 (6)	0.0056 (7)	0.0025 (6)
C3	0.0230 (9)	0.0267 (9)	0.0189 (8)	−0.0014 (7)	0.0072 (7)	0.0055 (7)
C4	0.0262 (10)	0.0352 (11)	0.0156 (8)	−0.0009 (7)	0.0091 (8)	0.0005 (7)
C5	0.0341 (10)	0.0256 (9)	0.0196 (8)	0.0000 (8)	0.0112 (8)	−0.0051 (7)
C6	0.0254 (9)	0.0185 (8)	0.0163 (8)	−0.0011 (7)	0.0069 (7)	−0.0007 (6)
C7	0.0122 (7)	0.0128 (7)	0.0137 (7)	−0.0002 (5)	0.0033 (6)	0.0007 (5)
C8	0.0153 (7)	0.0147 (7)	0.0115 (7)	0.0013 (6)	0.0032 (6)	−0.0003 (6)
C9	0.0160 (8)	0.0181 (8)	0.0226 (8)	−0.0014 (6)	0.0033 (7)	0.0017 (6)
C10	0.0176 (8)	0.0208 (8)	0.0218 (8)	0.0031 (6)	−0.0012 (7)	0.0006 (7)
C11	0.0286 (9)	0.0179 (8)	0.0152 (8)	0.0063 (7)	0.0057 (7)	0.0035 (6)
C12	0.0244 (9)	0.0198 (8)	0.0229 (9)	0.0012 (7)	0.0122 (7)	0.0047 (7)
C13	0.0174 (8)	0.0198 (8)	0.0211 (8)	0.0026 (6)	0.0084 (7)	0.0042 (6)
C14	0.0152 (8)	0.0189 (8)	0.0150 (8)	0.0013 (6)	0.0019 (6)	0.0043 (6)
C15	0.0149 (8)	0.0264 (9)	0.0146 (8)	0.0009 (6)	0.0011 (7)	0.0025 (6)
C16	0.0173 (8)	0.0139 (7)	0.0121 (7)	−0.0007 (6)	0.0033 (6)	0.0021 (6)
C17	0.0186 (8)	0.0201 (8)	0.0175 (8)	−0.0015 (6)	0.0069 (7)	−0.0007 (6)
C18	0.0275 (9)	0.0211 (9)	0.0190 (8)	0.0025 (7)	0.0114 (7)	−0.0015 (7)
C19	0.0359 (10)	0.0170 (8)	0.0148 (8)	−0.0040 (7)	0.0069 (7)	−0.0010 (6)
C20	0.0233 (9)	0.0213 (9)	0.0205 (8)	−0.0057 (7)	0.0027 (7)	0.0009 (7)
C21	0.0173 (8)	0.0178 (8)	0.0211 (8)	−0.0018 (6)	0.0053 (7)	0.0014 (6)
C22	0.0106 (7)	0.0204 (8)	0.0137 (7)	0.0017 (6)	0.0038 (6)	−0.0003 (6)
C23	0.0188 (8)	0.0223 (8)	0.0168 (8)	0.0005 (6)	0.0034 (7)	−0.0028 (6)
C24	0.0214 (9)	0.0371 (11)	0.0179 (8)	−0.0002 (8)	0.0025 (7)	−0.0078 (7)
C25	0.0199 (9)	0.0476 (12)	0.0131 (8)	0.0045 (8)	0.0033 (7)	0.0004 (7)
C26	0.0234 (9)	0.0335 (10)	0.0201 (9)	0.0031 (7)	0.0076 (7)	0.0087 (7)
C27	0.0190 (8)	0.0217 (8)	0.0192 (8)	0.0000 (6)	0.0056 (7)	0.0022 (6)
C28	0.0093 (7)	0.0152 (7)	0.0134 (7)	−0.0006 (5)	0.0029 (6)	−0.0010 (5)
C29	0.0163 (7)	0.0144 (7)	0.0137 (7)	−0.0015 (6)	0.0070 (6)	−0.0022 (6)
C30	0.0176 (8)	0.0194 (8)	0.0154 (7)	−0.0018 (6)	0.0059 (6)	−0.0014 (6)
C31	0.0287 (9)	0.0184 (8)	0.0161 (8)	0.0019 (7)	0.0080 (7)	0.0011 (6)
C32	0.0352 (10)	0.0159 (8)	0.0219 (8)	−0.0049 (7)	0.0154 (8)	−0.0025 (6)
C33	0.0239 (9)	0.0204 (8)	0.0260 (9)	−0.0083 (7)	0.0121 (8)	−0.0072 (7)
C34	0.0165 (8)	0.0187 (8)	0.0216 (8)	−0.0024 (6)	0.0062 (7)	−0.0034 (6)
C35	0.0136 (7)	0.0205 (8)	0.0150 (7)	0.0018 (6)	0.0051 (6)	−0.0020 (6)
C36	0.0143 (8)	0.0224 (8)	0.0187 (8)	0.0002 (6)	0.0066 (7)	0.0005 (6)
C37	0.0147 (7)	0.0135 (7)	0.0146 (7)	−0.0002 (6)	0.0071 (6)	0.0014 (6)
C38	0.0146 (8)	0.0175 (8)	0.0203 (8)	−0.0020 (6)	0.0048 (7)	−0.0006 (6)
C39	0.0159 (8)	0.0164 (8)	0.0284 (9)	0.0018 (6)	0.0105 (7)	0.0038 (7)
C40	0.0266 (9)	0.0147 (8)	0.0246 (9)	0.0022 (6)	0.0151 (8)	0.0002 (6)
C41	0.0252 (9)	0.0192 (8)	0.0183 (8)	−0.0017 (7)	0.0071 (7)	−0.0036 (6)
C42	0.0163 (8)	0.0198 (8)	0.0160 (8)	−0.0008 (6)	0.0039 (6)	−0.0009 (6)

### 1,1',1''-[(2-Iodoethoxy)methanetriyl]tribenzene (2) Geometric parameters (Å, º)

**Table:**

I1—C15	2.1555 (18)	C20—C21	1.388 (2)
I2—C36	2.1533 (18)	C20—H20	0.9500
O1—C14	1.4146 (19)	C21—H21	0.9500
O1—C7	1.4422 (19)	C22—C23	1.391 (2)
O2—C35	1.4227 (19)	C22—C27	1.399 (2)
O2—C28	1.4450 (18)	C22—C28	1.530 (2)
C1—C6	1.396 (2)	C23—C24	1.397 (2)
C1—C2	1.397 (2)	C23—H23	0.9500
C1—C7	1.531 (2)	C24—C25	1.382 (3)
C2—C3	1.392 (2)	C24—H24	0.9500
C2—H2	0.9500	C25—C26	1.388 (3)
C3—C4	1.384 (3)	C25—H25	0.9500
C3—H3	0.9500	C26—C27	1.385 (2)
C4—C5	1.389 (3)	C26—H26	0.9500
C4—H4	0.9500	C27—H27	0.9500
C5—C6	1.393 (2)	C28—C37	1.532 (2)
C5—H5	0.9500	C28—C29	1.539 (2)
C6—H6	0.9500	C29—C30	1.391 (2)
C7—C8	1.537 (2)	C29—C34	1.400 (2)
C7—C16	1.540 (2)	C30—C31	1.396 (2)
C8—C13	1.387 (2)	C30—H30	0.9500
C8—C9	1.395 (2)	C31—C32	1.383 (3)
C9—C10	1.390 (2)	C31—H31	0.9500
C9—H9	0.9500	C32—C33	1.389 (3)
C10—C11	1.386 (3)	C32—H32	0.9500
C10—H10	0.9500	C33—C34	1.386 (2)
C11—C12	1.386 (3)	C33—H33	0.9500
C11—H11	0.9500	C34—H34	0.9500
C12—C13	1.395 (2)	C35—C36	1.503 (2)
C12—H12	0.9500	C35—H35A	0.9900
C13—H13	0.9500	C35—H35B	0.9900
C14—C15	1.510 (2)	C36—H36A	0.9900
C14—H14A	0.9900	C36—H36B	0.9900
C14—H14B	0.9900	C37—C42	1.392 (2)
C15—H15A	0.9900	C37—C38	1.398 (2)
C15—H15B	0.9900	C38—C39	1.386 (2)
C16—C17	1.387 (2)	C38—H38	0.9500
C16—C21	1.400 (2)	C39—C40	1.388 (2)
C17—C18	1.399 (2)	C39—H39	0.9500
C17—H17	0.9500	C40—C41	1.384 (2)
C18—C19	1.381 (3)	C40—H40	0.9500
C18—H18	0.9500	C41—C42	1.392 (2)
C19—C20	1.388 (3)	C41—H41	0.9500
C19—H19	0.9500	C42—H42	0.9500
			
C14—O1—C7	117.95 (12)	C16—C21—H21	119.6
C35—O2—C28	116.86 (12)	C23—C22—C27	118.35 (16)
C6—C1—C2	118.05 (16)	C23—C22—C28	123.83 (15)
C6—C1—C7	119.94 (14)	C27—C22—C28	117.53 (14)
C2—C1—C7	121.71 (15)	C22—C23—C24	120.60 (17)
C3—C2—C1	120.95 (16)	C22—C23—H23	119.7
C3—C2—H2	119.5	C24—C23—H23	119.7
C1—C2—H2	119.5	C25—C24—C23	120.31 (18)
C4—C3—C2	120.47 (17)	C25—C24—H24	119.8
C4—C3—H3	119.8	C23—C24—H24	119.8
C2—C3—H3	119.8	C24—C25—C26	119.61 (17)
C3—C4—C5	119.23 (17)	C24—C25—H25	120.2
C3—C4—H4	120.4	C26—C25—H25	120.2
C5—C4—H4	120.4	C27—C26—C25	120.13 (18)
C4—C5—C6	120.36 (17)	C27—C26—H26	119.9
C4—C5—H5	119.8	C25—C26—H26	119.9
C6—C5—H5	119.8	C26—C27—C22	120.99 (17)
C5—C6—C1	120.92 (16)	C26—C27—H27	119.5
C5—C6—H6	119.5	C22—C27—H27	119.5
C1—C6—H6	119.5	O2—C28—C22	108.83 (12)
O1—C7—C1	109.02 (13)	O2—C28—C37	104.40 (12)
O1—C7—C8	104.00 (12)	C22—C28—C37	111.01 (13)
C1—C7—C8	112.74 (13)	O2—C28—C29	110.48 (13)
O1—C7—C16	110.61 (13)	C22—C28—C29	114.04 (13)
C1—C7—C16	112.88 (13)	C37—C28—C29	107.65 (13)
C8—C7—C16	107.23 (13)	C30—C29—C34	118.49 (15)
C13—C8—C9	118.53 (15)	C30—C29—C28	121.09 (14)
C13—C8—C7	121.16 (14)	C34—C29—C28	120.30 (15)
C9—C8—C7	120.27 (14)	C29—C30—C31	120.70 (16)
C10—C9—C8	120.75 (16)	C29—C30—H30	119.7
C10—C9—H9	119.6	C31—C30—H30	119.7
C8—C9—H9	119.6	C32—C31—C30	120.18 (17)
C11—C10—C9	120.30 (16)	C32—C31—H31	119.9
C11—C10—H10	119.8	C30—C31—H31	119.9
C9—C10—H10	119.8	C31—C32—C33	119.56 (16)
C10—C11—C12	119.35 (16)	C31—C32—H32	120.2
C10—C11—H11	120.3	C33—C32—H32	120.2
C12—C11—H11	120.3	C34—C33—C32	120.36 (16)
C11—C12—C13	120.25 (16)	C34—C33—H33	119.8
C11—C12—H12	119.9	C32—C33—H33	119.8
C13—C12—H12	119.9	C33—C34—C29	120.68 (16)
C8—C13—C12	120.80 (16)	C33—C34—H34	119.7
C8—C13—H13	119.6	C29—C34—H34	119.7
C12—C13—H13	119.6	O2—C35—C36	107.63 (13)
O1—C14—C15	107.16 (13)	O2—C35—H35A	110.2
O1—C14—H14A	110.3	C36—C35—H35A	110.2
C15—C14—H14A	110.3	O2—C35—H35B	110.2
O1—C14—H14B	110.3	C36—C35—H35B	110.2
C15—C14—H14B	110.3	H35A—C35—H35B	108.5
H14A—C14—H14B	108.5	C35—C36—I2	112.77 (11)
C14—C15—I1	112.97 (12)	C35—C36—H36A	109.0
C14—C15—H15A	109.0	I2—C36—H36A	109.0
I1—C15—H15A	109.0	C35—C36—H36B	109.0
C14—C15—H15B	109.0	I2—C36—H36B	109.0
I1—C15—H15B	109.0	H36A—C36—H36B	107.8
H15A—C15—H15B	107.8	C42—C37—C38	118.56 (15)
C17—C16—C21	118.45 (15)	C42—C37—C28	121.26 (14)
C17—C16—C7	121.31 (14)	C38—C37—C28	120.15 (14)
C21—C16—C7	120.13 (14)	C39—C38—C37	120.47 (16)
C16—C17—C18	120.71 (16)	C39—C38—H38	119.8
C16—C17—H17	119.6	C37—C38—H38	119.8
C18—C17—H17	119.6	C38—C39—C40	120.51 (16)
C19—C18—C17	120.26 (16)	C38—C39—H39	119.7
C19—C18—H18	119.9	C40—C39—H39	119.7
C17—C18—H18	119.9	C41—C40—C39	119.47 (16)
C18—C19—C20	119.52 (16)	C41—C40—H40	120.3
C18—C19—H19	120.2	C39—C40—H40	120.3
C20—C19—H19	120.2	C40—C41—C42	120.17 (16)
C21—C20—C19	120.29 (17)	C40—C41—H41	119.9
C21—C20—H20	119.9	C42—C41—H41	119.9
C19—C20—H20	119.9	C41—C42—C37	120.80 (16)
C20—C21—C16	120.72 (16)	C41—C42—H42	119.6
C20—C21—H21	119.6	C37—C42—H42	119.6
			
C6—C1—C2—C3	0.2 (3)	C27—C22—C23—C24	0.8 (3)
C7—C1—C2—C3	173.91 (16)	C28—C22—C23—C24	174.58 (16)
C1—C2—C3—C4	0.5 (3)	C22—C23—C24—C25	−0.7 (3)
C2—C3—C4—C5	−0.4 (3)	C23—C24—C25—C26	0.2 (3)
C3—C4—C5—C6	−0.3 (3)	C24—C25—C26—C27	0.1 (3)
C4—C5—C6—C1	1.0 (3)	C25—C26—C27—C22	0.0 (3)
C2—C1—C6—C5	−0.9 (3)	C23—C22—C27—C26	−0.5 (3)
C7—C1—C6—C5	−174.73 (16)	C28—C22—C27—C26	−174.66 (15)
C14—O1—C7—C1	50.77 (17)	C35—O2—C28—C22	46.44 (17)
C14—O1—C7—C8	171.25 (13)	C35—O2—C28—C37	165.03 (13)
C14—O1—C7—C16	−73.92 (17)	C35—O2—C28—C29	−79.50 (16)
C6—C1—C7—O1	80.01 (18)	C23—C22—C28—O2	−108.95 (17)
C2—C1—C7—O1	−93.57 (18)	C27—C22—C28—O2	64.84 (18)
C6—C1—C7—C8	−34.9 (2)	C23—C22—C28—C37	136.69 (16)
C2—C1—C7—C8	151.47 (15)	C27—C22—C28—C37	−49.52 (19)
C6—C1—C7—C16	−156.65 (15)	C23—C22—C28—C29	14.9 (2)
C2—C1—C7—C16	29.8 (2)	C27—C22—C28—C29	−171.32 (14)
O1—C7—C8—C13	12.82 (19)	O2—C28—C29—C30	−8.2 (2)
C1—C7—C8—C13	130.77 (16)	C22—C28—C29—C30	−131.12 (15)
C16—C7—C8—C13	−104.38 (17)	C37—C28—C29—C30	105.25 (16)
O1—C7—C8—C9	−169.74 (14)	O2—C28—C29—C34	175.75 (14)
C1—C7—C8—C9	−51.8 (2)	C22—C28—C29—C34	52.8 (2)
C16—C7—C8—C9	73.05 (18)	C37—C28—C29—C34	−70.83 (18)
C13—C8—C9—C10	1.5 (3)	C34—C29—C30—C31	−1.6 (2)
C7—C8—C9—C10	−176.04 (15)	C28—C29—C30—C31	−177.75 (15)
C8—C9—C10—C11	−1.2 (3)	C29—C30—C31—C32	1.5 (3)
C9—C10—C11—C12	0.1 (3)	C30—C31—C32—C33	−0.4 (3)
C10—C11—C12—C13	0.7 (3)	C31—C32—C33—C34	−0.6 (3)
C9—C8—C13—C12	−0.6 (2)	C32—C33—C34—C29	0.5 (3)
C7—C8—C13—C12	176.84 (15)	C30—C29—C34—C33	0.6 (2)
C11—C12—C13—C8	−0.5 (3)	C28—C29—C34—C33	176.76 (15)
C7—O1—C14—C15	−179.79 (13)	C28—O2—C35—C36	−177.23 (13)
O1—C14—C15—I1	−58.47 (15)	O2—C35—C36—I2	−63.50 (15)
O1—C7—C16—C17	−13.9 (2)	O2—C28—C37—C42	20.69 (19)
C1—C7—C16—C17	−136.35 (16)	C22—C28—C37—C42	137.79 (15)
C8—C7—C16—C17	98.89 (17)	C29—C28—C37—C42	−96.74 (17)
O1—C7—C16—C21	169.95 (14)	O2—C28—C37—C38	−161.21 (14)
C1—C7—C16—C21	47.5 (2)	C22—C28—C37—C38	−44.10 (19)
C8—C7—C16—C21	−77.26 (18)	C29—C28—C37—C38	81.36 (18)
C21—C16—C17—C18	−1.9 (2)	C42—C37—C38—C39	−0.1 (2)
C7—C16—C17—C18	−178.14 (15)	C28—C37—C38—C39	−178.25 (15)
C16—C17—C18—C19	2.0 (3)	C37—C38—C39—C40	−0.7 (3)
C17—C18—C19—C20	−0.2 (3)	C38—C39—C40—C41	0.3 (3)
C18—C19—C20—C21	−1.7 (3)	C39—C40—C41—C42	0.9 (3)
C19—C20—C21—C16	1.7 (3)	C40—C41—C42—C37	−1.7 (3)
C17—C16—C21—C20	0.1 (2)	C38—C37—C42—C41	1.3 (2)
C7—C16—C21—C20	176.33 (15)	C28—C37—C42—C41	179.42 (15)
